# P-1627. Impact of Maternal SARS-CoV-2 Infection on the Risk of Developing Preeclampsia and Eclampsia: A Retrospective Cohort Study with Variant-Specific Analysis

**DOI:** 10.1093/ofid/ofaf695.1803

**Published:** 2026-01-11

**Authors:** Muzna Lone, Atika Jabeen, Syeda Fatima Shariq, Jameela Ali Al Ajmi, Adeel A Butt

**Affiliations:** Hamad Medical Corporation, Doha, Ar Rayyan, Qatar; Hamad Medical Corporation, Doha, Ar Rayyan, Qatar; Hamad Medical Corporation, Doha, Ar Rayyan, Qatar; Hamad Medical Corporation, Doha, Ar Rayyan, Qatar; Hackensack Meridian JFK University Medical Center , Edison, NJ

## Abstract

**Background:**

The relationship between maternal SARS-CoV-2 infection and the risk of hypertensive disorders of pregnancy, including preeclampsia and eclampsia, remains uncertain. This study explores the impact of maternal SARS-CoV-2 infection on the risk of preeclampsia/eclampsia, with a focus on variant specific-risks.

**Figure:**
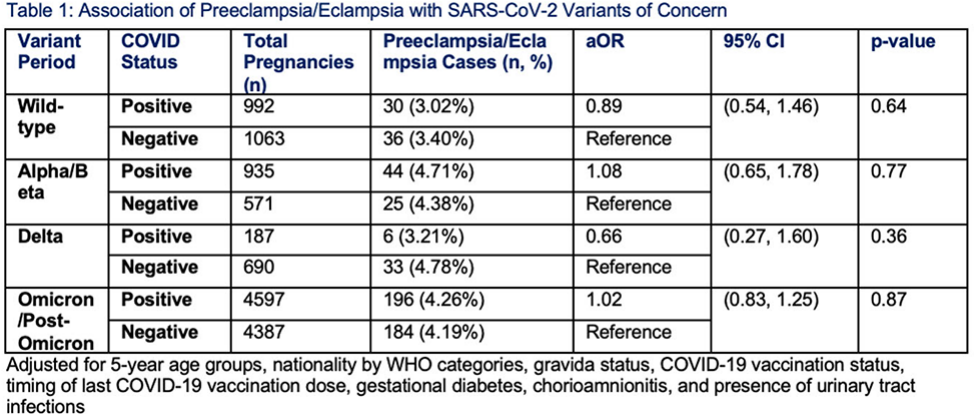

**Figure:**
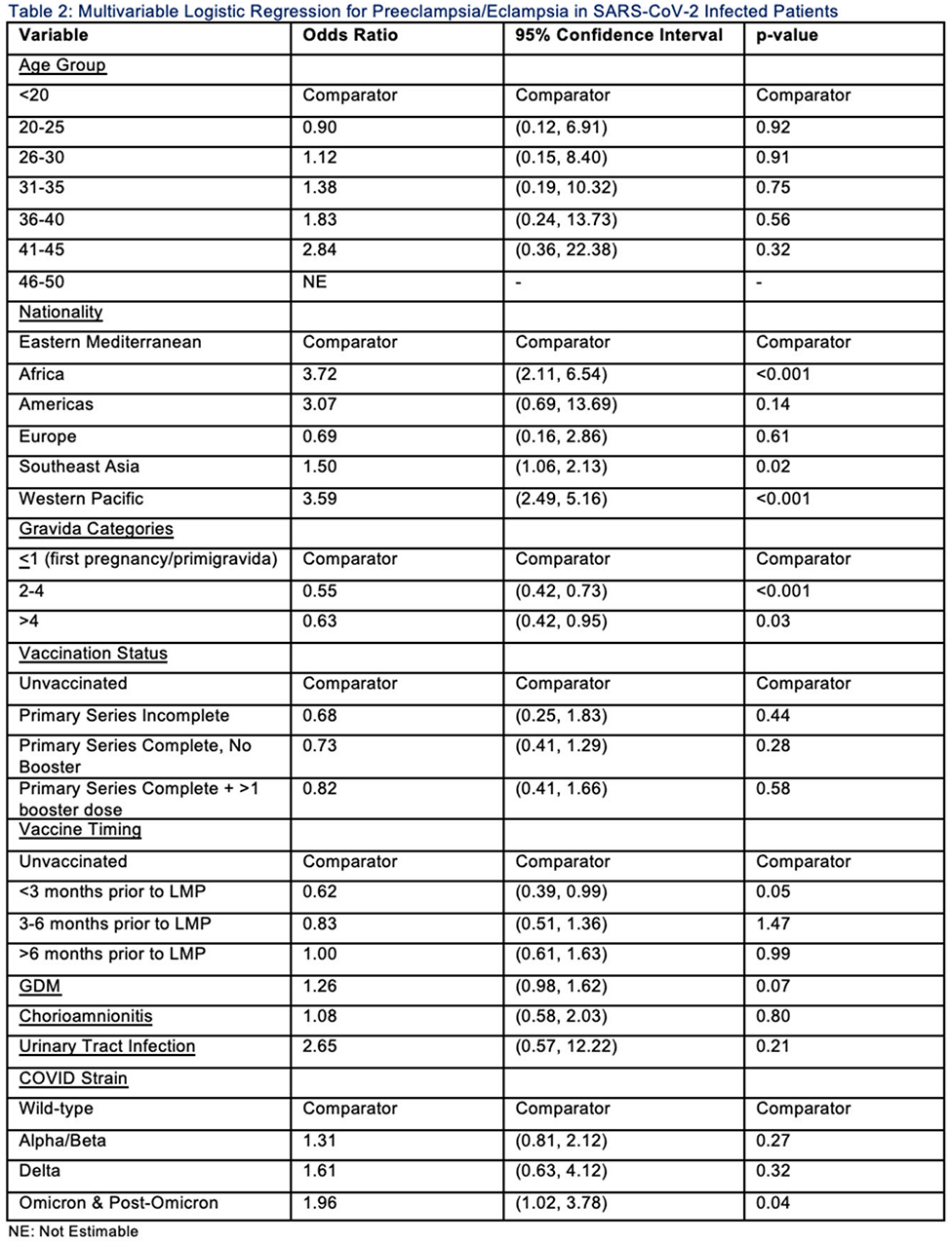

**Methods:**

We conducted a retrospective cohort study at Hamad Medical Corporation (HMC) in Qatar, which manages the majority of the country’s obstetric cases. Data on all pregnant patients from January 2020 to May 2024 were retrieved from electronic medical records, and singleton pregnancies with at least one RT-PCR COVID-19 test during pregnancy were included. COVID-positive pregnancies were matched 1:1 to COVID-negative pregnancies (>1 negative test, no positive tests) based on 5-year age groups, WHO nationality categories, gravida status, COVID-19 test timing, and vaccination status. Variant predominant periods were classified as Wild-type, Alpha/Beta, Delta, and Omicron/Post-Omicron. Multivariable logistic regression was used to calculate adjusted odds ratios (aORs) and 95% confidence intervals (CIs) for associations between SARS-CoV-2 variant predominant periods and preeclampsia/eclampsia. Additional regression analysis was performed to assess associations within the COVID-positive group.

**Results:**

A total of 6711 COVID-positive and 6711 COVID-negative women with singleton pregnancies were identified and matched. In the overall cohort, there were no significant differences in preeclampsia/eclampsia risk across variant periods. Among COVID-positive pregnancies, infection during the Omicron/Post-Omicron predominant period was significantly associated with higher odds of preeclampsia/eclampsia (aOR: 1.96, 95% CI: 1.02-3.78, p=0.04).

**Conclusion:**

Maternal SARS-CoV-2 infection during the Omicron/Post-Omicron period was associated with an increased risk of preeclampsia/eclampsia. This finding underscores the need for enhanced monitoring/screening based in targeted groups of pregnant women.

**Disclosures:**

All Authors: No reported disclosures

